# Scintigraphy evaluation of hyperthyroidism and its correlation with clinical and biochemical profiles

**DOI:** 10.1186/s13104-020-05164-5

**Published:** 2020-07-06

**Authors:** Khaled Alswat, Sara Ahmad Assiri, Raad M. M. Althaqafi, Atheer Alsufyani, Abaad Althagafi, Sara Alrebaiee, Najd Alsukhayri

**Affiliations:** 1 Department of Medicine, Taif University School of Medicine, Taif, Saudi Arabia; 2 Taif University School of Medicine, Taif, Saudi Arabia

**Keywords:** Hyperthyroidism, Thyrotoxicosis, Graves’ disease, Thyroid uptake, Scintigraphy

## Abstract

**Objective:**

Hyperthyroidism is the excessive synthesis of thyroid hormones. Thyroid uptake scans and ultrasonography provide an accurate diagnosis of hyperthyroidism, especially when thyroid receptor antibody (TRAb) measurement is not readily available. This study explored the prevalence of various hyperthyroidism causes using retrospective scintigraphy results and evaluated their relationship with clinical, biochemical, and sonographic imaging parameters from patients who underwent ^99m^Tc-pertechnetate thyroid scans between 2016 and 2019 in Taif, Saudi Arabia, where literature is insufficient. Furthermore, the inappropriate use of thyroid scanning in different thyroid diseases was evaluated.

**Results:**

The study enrolled 207 patients (mean age: 42.5 ± 14.7 years). The mean free T4, T3, antithyroid peroxidase antibody, antithyroglobulin antibody, C-reactive protein, and erythrocyte sedimentation rate levels were high. Graves’ disease was the most common diagnosis. Compared to toxic solitary/multinodular goiter, patients with Graves’ disease were usually younger, used carbimazole during both the uptake and the scan, had an enlarged thyroid gland, and had higher FT4 and FT3 levels. Inappropriate thyroid uptake and scan use was reported in approximately 10% of patients, and 25% of the patients used carbimazole during the uptake and scan. Thus, better patient education is needed to avoid misinterpreting the scan results.

## Introduction

Thyrotoxicosis can occur in hyperthyroidism following the release of a pre-synthesized thyroid hormone or from an extrathyroidal thyroid hormone source [1]. Hyperthyroidism is usually caused by Graves’ disease (GD) and toxic multinodular goiter (TMNG), and occasionally caused by thyroiditis [2, 3]. The accurate diagnosis of GD from thyroiditis is crucial to avoid prescribing antithyroid medications to thyroiditis patients, which could lead to hypothyroidism or other side-effects [4].

Furthermore, treatment of TMNG with antithyroid medications is discouraged; rather, radioiodine or surgery is the intervention of choice. This highlights the importance of distinguishing between TMNG and GD patients when the clinical examination is inconclusive [5]. GD is an autoimmune disease caused by circulating thyroid receptor antibodies (TRAb), which are disease hallmarks of GD [6]. Measuring antibody levels can supplement clinical characteristics to provide a rapid and accurate diagnosis of GD [7].

When the TRAb test is unavailable and the patient presents with an atypical clinical GD picture, thyroid scintigraphy and peroxidase antibody measurement can be sufficient to differentiate GD from other causes of thyrotoxicosis. Thyroid uptake and scans help to differentiate between productive and destructive thyrotoxicosis, and between diffuse and focal overactivity [8]. The accuracy of radionuclide thyroid scanning is compromised by antithyroid medications such as carbimazole and propyluracil; thus, these medications should be discontinued prior to scanning in order to optimize the efficiency of the scan and increase diagnostic accuracy [9]. One contraindication to thyroid scanning is pregnancy and lactation [10]. Ultrasonography is a readily available tool to differentiate between different causes of thyrotoxicosis [11, 12].

The limited availability of the TRAb test necessitates the use of alternatives to guide clinical decisions in cases of unclear presentation. The current study explores the profiles of a sample of hyperthyroidism patients for which the TRAb test was not available. Thus, various causes of hyperthyroidism and their prevalence in Taif City, Saudi Arabia—where literature is insufficient—were analyzed using scintigraphy results. The relationship of the final diagnosis to the clinical, biochemical, and sonographic imaging parameters were also assessed. In addition, this study evaluated the inappropriate use of the thyroid scanning in different thyroid diseases.

## Main text

### Patients and methods

We retrospectively reviewed the medical records of patients who underwent a ^99m^Tc-pertechnetate thyroid scan to evaluate for thyroid dysfunction as clinically indicated by their healthcare provider. Hyperthyroidism status was determined on the basis of the patient’s scintigraphy data and on clinical presentation, biochemical data, and sonographic imaging. Researchers reviewed all thyroid scans that were performed between 2016 and 2019 in Al-Hada Armed Forces Hospital in Taif, Saudi Arabia. Patients of all ages who underwent ^99m^Tc-pertechnetate thyroid scanning during the indicated period were included. Patients were excluded if thyroid scans were performed to investigate previous thyroid operations (e.g., lobectomy), if the data were incomplete, or if relevant radiological reports or medical records were missing.

### Data collection

Data from 207 patients were collected and recorded on an Excel spreadsheet. Age, gender, presenting symptoms of hyperthyroidism at the time of referral for thyroid uptake scan, and comorbid conditions were recorded. Furthermore, all laboratory data obtained within 1 month prior to the thyroid scan referral were collected, and medications such as carbimazole, propranolol, and amiodarone were also documented. We also collected data from the thyroid ultrasound that was performed on all patients who had a thyroid uptake scan within 1 month of the scan date. Thyroid ultrasound features including texture, vascularity, and lymph node enlargement were collected from the radiologist’s report.

### Statistical analysis

Data were analyzed with SPSS v. 20. Qualitative data variables were expressed as frequencies and percentages, while quantitative variables were expressed as means and standard deviations (SD). An independent sample *t* test was used to identify significant differences between variables, and *p*-values ≤0.05 were considered significant.

## Results

The review of 207 thyroid uptake scan results indicated a mean patient age of 42.5 ± 14.7 years, with most patients being female. One-hundred-and-sixty patients underwent a thyroid ultrasound, and most had a normal thyroid size and vascularity, but had a heterogenous gland. The most common presenting symptoms were eye manifestations, palpitations, tremor, and neck swelling, while the least common were nausea, vomiting, insomnia, and heat intolerance. The most frequently reported comorbid conditions were type II diabetes, hypertension, and dermatitis. Approximately 15% of patients reported a recent history of upper respiratory tract infection at the time of the thyroid uptake and scan (Table 1). Approximately 25% of patients reported using carbimazole both before and during the thyroid uptake and scan, whereas only 10% used it only after completing the thyroid uptake and scan. The use of carbimazole during the scan was highest in GD patients, 13.2% of whom had normal scan results despite having active GD (Additional file 1: Figure S1).

**Table 1:** Baseline characteristics of the whole cohort

Baseline characteristics (N = 207)		Thyroid ultrasound (N = 160)	
Mean age (years)	42.5 + 14.7	Normal (%)	63.8
Female (%)	75.8	Enlarged (%)	36.2
Presenting symptoms		Thyroid ultrasound texture	
Dysphagia and/or hoarseness (%)	3.4	Heterogenous (%)	73.1
Neck swelling (%)	6.3	Homogenous (%)	26.9
Neck tenderness (%)	1.9	Thyroid ultrasound vascularity	
Weight loss (%)	7.7	Normal (%)	54.4
Tremor (%)	6.8	Hypervascular (%)	43.8
Palpitation (%)	7.7	Hypovascular (%)	1.8
Fatigue (%)	4.8	Lymph node features in the thyroid ultrasound	
Anxiety (%)	3.4	No lymph node enlargement (%)	62.5
Insomnia (%)	1.0	Bilateral lymph node enlargement (%)	35
Headache (%)	2.4	Unilateral lymph node enlargement (%)	2.5
Nausea and/or vomiting (%)	1.0	Multiple lymph node enlargement (%)	43.7
Eye manifestations (%)	13.5	Single lymph node enlargement (%)	17.5
Heat intolerance (%)	1.9	Thyroid uptake scan (N = 207)	
Comorbidities		Heterogenous (%)	37.4
Upper respiratory tract infection (%)	15.0	Homogenous (%)	55.3
Hypertension (%)	15.0	Not visualized (%)	7.3
Hyperlipidemia (%)	11.1	Mean uptake (%)	19.1 + 14.4%
Type II Diabetes (%)	19.3	Diagnosis based on the thyroid uptake scan result	
Type I diabetes (%)	2.4	Graves’ disease (%)	25.6
Anemia (%)	8.7	Thyroiditis (%)	15.9
Dermatitis (%)	15.0	Normal (%)	4.3
Ischemic heart disease (%)	1.4	Normal scan while taking carbimazole (%)	14
Stroke (%)	1.4	Autonomous nodule (%)	4.3
Deep vein thrombosis and/or pulmonary embolism (%)	1.4	Toxic multi-nodular goiter (%)	37.2
Medications		Simple goiter (%)	6.3
Carbimazole usage prior to the thyroid scan (%)	23.7	Nodular goiter with cold nodule (%)	1.0
Carbimazole usage after the thyroid scan (%)	10.6	Cold nodule (%)	1.0
Carbimazole usage during the thyroid scan (%)	25.1	Toxic multi-nodular goiter with a cold nodule (%)	2.4
Propranolol (%)	29.0	Marine-Lenhart syndrome (%)	1.0
Levothyroxine (%)	8.7	Recurrnt hyperthyroidism after surgical resection (%)	1.0
Amiodarone (%)	0.5	Year of the nuclear scan	
Artificial eye tears (%)	5.8	2019	8.2
Laboratory data		2018	22.2
TSH (milli-international units per liter)	1.2 + 8.5	2017	26.1
Free T4 (pmol/L)	20.3 + 10.0	2016	43.5
Free T3 (pmol/L)	9.8 + 8.8	Biochemical diagnosis	
Antithyroid peroxidase antibody (IU/mL)	322.2 + 531.8	Subclinical hyperthyroidism	42.5
Antithyroglobulin antibody (IU/mL)	336.2 + 901.1	Hyperthyroidism	43.5
Vitamin D (ng/mL)	22.0 + 9.8	Euthyroidism	8.2
Erythrocyte sedimentation rate (ESR) (mm/hr)	32.5 + 22.8	T3 thyro-toxicosis	1.4
C-Reactive protein (CRP) (mg/L)	10.0 + 10.9	Hypothyroidism	1.4
		Subclinical hypothyroidism	1.9

This table shows the baseline characteristics of the whole cohort using the means and standard deviations for quantitative variables like the age, thyroid uptake scan and laboratory parameter. Percentages were used to express all other qualitative data

Propranolol was used in approximately one-third of patients. The mean thyroid stimulating hormone (TSH) levels fell in the normal range, while mean free T4, T3, antithyroid peroxidase antibody, antithyroglobulin antibody, CRP, and ESR values were high. However, mean vitamin D levels fell in the insufficient range. The most likely biochemical diagnosis was clinical or subclinical hyperthyroidism.

The majority of the thyroid ultrasound results showed no lymphadenopathy; however, those with lymphadenopathy showed evidence of bilateral and multiple lymph node enlargement. The thyroid uptake and scan mainly showed homogenous high uptake, and GD was the most common diagnosis, followed by a TMNG. Furthermore, the results revealed a decreasing percentage of thyroid uptake and scans ordered; 2016 was the year with the most scans, followed by 2017, 2018, and then 2019 (Additional file 1: Figure S2).

Relative to subclinical hyperthyroidism, patients with clinical hyperthyroidism were more likely to be younger (P = 0.060); have weight loss (P = 0.016), tremor (P = 0.044), or palpitations (P = 0.003); use carbimazole during the thyroid uptake and scan; use propranolol (P = 0.004 and P = 0.007, respectively); have lower TSH and higher FT4 and FT3 (all P < 0.001); have a homogenous gland in the thyroid uptake and scan (P = 0.006); and to have been diagnosed with GD and autonomous thyroid nodules (P = 0.002; Table 2). Those with subclinical hyperthyroidism showed a non-significant trend of increased incidence of hypertension, stroke, and heart disease relative to those with clinical hyperthyroidism.

**Table 2:** Comparison based on the biochemical diagnosis

Variables	Hyperthyroidism	Subclinical hyperthyroidism	P value
Number	90	88	n/a
Mean age (years)	41.0 + 13.5	45.1 + 15.6	0.060
Female (%)	73.3	75	0.467
Presenting symptoms			
Dysphagia and/or hoarseness (%)	2.2	5.7	0.213
Neck swelling (%)	7.8	4.6	0.281
Neck tenderness (%)	4.4	0.0	0.063
Weight loss (%)	13.3	3.4	0.016
Tremor (%)	11.1	3.4	0.044
Palpitation (%)	14.4	2.3	0.003
Fatigue (%)	8.9	2.3	0.054
Anxiety (%)	3.3	3.4	0.648
Insomnia (%)	2.2	0.0	0.254
Headache (%)	3.3	2.3	0.511
Nausea and/or vomiting (%)	1.1	1.1	0.746
Eye manifestations (%)	14.4	12.5	0.437
Heat intolerance (%)	2.2	2.3	0.681
Comorbidities			
Upper respiratory tract infection (%)	13.3	13.6	0.563
Hypertension (%)	12.2	19.3	0.137
Hyperlipidemia (%)	11.1	9.1	0.422
Type II Diabetes (%)	14.4	26.1	0.039
Type I diabetes (%)	2.2	1.1	0.508
Anemia (%)	5.6	12.5	0.087
Dermatitis (%)	15.6	14.8	0.525
Ischemic heart disease (%)	0.0	2.3	0.243
Stroke (%)	0.0	2.3	0.243
Deep vein thrombosis and/or pulmonary embolism (%)	2.2	1.1	0.508
Medications			
Carbimazole usage prior to the thyroid scan (%)	20.5	30.7	0.071
Carbimazole usage after the thyroid scan (%)	14.4	10.2	0.266
Carbimazole usage during the thyroid scan (%)	35.6	17.1	0.004
Propranolol (%)	41.1	22.7	0.007
Amiodarone (%)	0.0	1.1	0.494
Artificial eye tears (%)	6.7	4.6	0.388
Laboratory data			
TSH (milli-international units per liter)	0.019 + 0.04	0.083 + 0.10	< 0.001
Free T4 (pmol/L)	28.7 + 9.9	14.5 + 2.4	< 0.001
Free T3 (pmol/L)	15.0 + 10.7	5.2 + 1.2	< 0.001
Antithyroid peroxidase antibody (IU/mL)	137.2 + 228.9	736.2 + 1495.5	0.309
Antithyroglobulin antibody (IU/mL)	140.6 + 185.1	384.7 + 657.8	0.161
Vitamin D (ng/mL)	23.4 + 10.4	22.4 + 9.0	0.289
Erythrocyte sedimentation rate (ESR) (mm/hr)	34.5 + 24.3	34.4 + 24.7	0.995
C-Reactive protein (CRP) (mg/L)	9.8 + 12.4	11.3 + 9.3	0.719
Thyroid ultrasound			
Enlarged thyroid gland (%)	25.0	31.1	0.605
Heterogenous (%)	52.2	61.4	0.337
Hypervascular (%)	38.9	31.8	0.392
Bilateral lymph node enlargement (%)	22.2	33.0	0.404
Unilateral lymph node enlargement (%)	2.2	1.1	
Multiple lymph node enlargement (%)	27.8	38.6	0.093
Single lymph node enlargement (%)	10.0	15.9	
Thyroid uptake and scan			
Heterogenous (%)	26.7	48.9	0.006 0.006
Homogenous (%)	66.7	43.2	
Mean uptake (%)	17.6 + 9.5	13.2 + 3.8	0.373
Graves’ disease (%)	40	13.6	0.002
Thyroiditis (%)	8.9	27.3	
Normal (%)	2.2	5.7	
Autonomous nodule (%)	6.7	3.4	
Toxic multi-nodular goiter (%)	34.4	40.9	
Simple goiter (%)	4.4	4.5	
Nodular goiter with cold nodule (%)	0.0	2.2	
Cold nodule (%)	1.1	1.1	
Toxic multi-nodular goiter with a cold nodule (%)	1.1	1.1	
Marine-Lenhart syndrome (%)	2.2	0.0	
Normal scan while taking carbimazole (%)	17.8	11.4	0.159

This Table shows a comparison based on the biochemical diagnosis between Hyperthyroidism and Subclinical hyperthyroidism patients. The means and standard deviations are used for quantitative variables like the age, thyroid uptake scan and laboratory parameter. Percentages are used to express all other qualitative data. An independent sample t-test is used to identify significant differences between variables, and p-values ≤0.05 are considered significant

Compared to patients with a toxic solitary/multinodular goiter, those with GD (Table 3) were more likely to be younger (P = 0.001), use carbimazole during the thyroid uptake and scan (P = 0.001), use propranolol (P = 0.010), have higher FT4 and FT3 levels (P = 0.005 and P = 0.0032, respectively), have an enlarged thyroid gland on the thyroid ultrasound (P = 0.591), have homogenous thyroid uptake and scan (P < 0.001), and have clinical hyperthyroidism (P = 0.039); they were less likely to have lymphadenopathy (P = 0.008), type II diabetes (P = 0.009), and cardiovascular disease (P = 0.619).

**Table 3:** Comparison based on thyroid uptake scans results

Variables	Grave’s disease	Toxic solitary/multinodular goiter	P value
Number	53	86	n/a
Mean age (years)	35.8 + 11.1	43.7 + 14.6	0.001
Female (%)	73.6	77.9	0.351
Presenting symptoms			
Dysphagia and/or hoarseness (%)	3.8	3.5	0.633
Neck swelling (%)	1.9	7.0	0.178
Neck tenderness (%)	0.0	2.3	0.381
Weight loss (%)	9.4	4.7	0.222
Tremor (%)	13.2	5.8	0.117
Palpitation (%)	7.5	5.8	0.471
Fatigue (%)	7.5	2.3	0.149
Anxiety (%)	5.7	0.0	0.053
Insomnia (%)	0.0	2.3	0.381
Headache (%)	3.8	1.2	0.324
Nausea and/or vomiting (%)	1.9	0.0	0.381
Eye manifestations (%)	13.2	15.1	0.481
Heat intolerance (%)	1.9	2.3	0.676
Comorbidities			
Upper respiratory tract infection (%)	9.4	17.4	0.145
Hypertension (%)	5.7	16.3	0.052
Hyperlipidemia (%)	3.8	9.3	0.190
Type II Diabetes (%)	7.5	24.4	0.009
Type I diabetes (%)	3.8	1.2	0.324
Anemia (%)	9.4	5.8	0.316
Dermatitis (%)	9.4	15.1	0.242
Ischemic heart disease (%)	0.0	1.2	0.619
Stroke (%)	0.0	1.3	0.619
Deep vein thrombosis and/or pulmonary embolism (%)	0.0	2.3	0.381
Medications			
Carbimazole usage prior to the thyroid scan (%)	28.3	30.2	0.482
Carbimazole usage after the thyroid scan (%)	17.0	10.5	0.196
Carbimazole usage during the thyroid scan (%)	47.2	20.9	0.001
Normal thyroid uptake&scan while taking carbimazole (%)	13.2	15.1	0.481
Propranolol (%)	49.1	27.9	0.010
Artificial eye tears (%)	5.7	7.0	0.529
Laboratory data			
TSH (milli-international units per liter)	0.28 + 1.1	0.31 + 1.4	0.883
Free T4 (pmol/L)	26.0 + 13.2	20.1 + 8.9	0.005
Free T3 (pmol/L)	14.5 + 11.7	9.6 + 7.5	0.032
Antithyroid peroxidase antibody (IU/mL)	216.4 + 287.0	251.4 + 500.2	0.831
Antithyroglobulin antibody (IU/mL)	503.0 + 1009.0	355.6 + 1131.0	0.723
Vitamin D (ng/mL)	21.7 + 11.1	21.6 + 9.2	0.967
Erythrocyte sedimentation rate (ESR) (mm/hr)	29.3 + 20.4	33.0 + 22.9	0.697
C-Reactive protein (CRP) (mg/L)	8.3 + 11.3	9.0 + 12.0	0.898
Thyroid ultrasound			
Enlarged thyroid gland (%)	32.1	25.6	0.591
Heterogenous (%)	49.1	60.5	0.306
Hypervascular (%)	39.6	40.7	0.976
Bilateral lymph node enlargement (%)	18.9	30.2	0.297
Unilateral lymph node enlargement (%)	1.9	0.0	
Multiple lymph node enlargement (%)	15.1	38.4	0.008
Single lymph node enlargement (%)	13.2	15.1	
Thyroid uptake and scan			
Heterogenous (%)	3.8	57.0	< 0.001
Homogenous (%)	96.2	43.0	
Mean uptake (%)	19.7 + 16.2	16.0 + 6.4	0.598
Biochemical diagnosis			
Subclinical hyperthyroidism	22.6	45.3	0.039
Hyperthyroidism	67.9	43.0	
Euthyroidism	3.8	9.3	
T3 thyro-toxicosis	1.9	1.2	
Hypothyroidism	1.9	0.0	
Subclinical hypothyroidism	1.9	1.2	

This table shows a comparison based on thyroid uptake scans results between Grave’s disease and Toxic solitary/multinodular goiter. The means and standard deviations are used for quantitative variables like the age, thyroid uptake scan and laboratory parameter. Percentages are used to express all other qualitative data. An independent sample t-test is used to identify significant differences between variables, and p-values ≤0.05 are considered significant

## Discussion

The prevalence of hyperthyroidism in the current study was three-fold higher in females (75%) than in males, and the mean age was 42.5 ± 14.7 years. These results are similar to those of a previous study in Riyadh, which found that over two-thirds of GD patients were female and that the mean age at diagnosis was 32 ± 0.9 years [13]. The high prevalence among women, specifically those with GD, could be attributed to many factors, such as a high genetic susceptibility, human leukocyte antigen (HLA) alleles (e.g., the HLA-B*46 allele in the Asian population), and the influence of estrogen on the immune system, particularly on B cells [14–16].

Indeed, the incidence of GD was reported to be five times higher in females and was found to occur predominantly during their reproductive years [17]. Environmental conditions also play an important role; for example, living at a high altitude is associated with elevated T3/T4 levels without an accompanied increase in TSH [18]. Slight hyperthyroidism is thought to be necessary to overcome the effect of high altitude-induced hypoxia because thyroid hormones are responsible for 2,3-diphosphoglycerate induction in red blood cells to facilitate oxygen release to the tissues [19]. The current biochemical results in the GD group show elevated T3/T4 levels with suppressed TSH, which is consistent with the results of Usman et al.’s study [13].

Elevated anti-thyroid peroxidase (anti-TPO) antibody levels were present in all GD patients in our study, which suggests that elevated anti-TPO may be a potentially useful diagnostic marker of GD. The importance of the anti-TPO marker is highlighted when diagnosing the subclinical form of GD in those at risk of developing clinical GD before the appearance of clinical manifestations, especially when an increase in anti-TPO is associated with nuclear uptake of > 0.4%, which was encountered in this study and corroborated the results of a recent study [20].

Differentiation between subclinical GD and thyroiditis based on clinical manifestations can be challenging. In such conditions, an isotope uptake scan of the thyroid is a definitive diagnostic tool [21]. Evaluation by ^99m^Tc-pertechnetate scintigraphy is used as a noninvasive method to investigate hyperthyroidism and has the advantage of short retention in the gland and absence of β-radiation [22]. Carbimazole should be discontinued for at least seven days before the scan because it interferes with the scan results. Our study found that 25% of patients continued to use carbimazole during the scan, which can alter the accuracy of the results [23]. This could result from physician miscommunication with the patient or from a lack of knowledge. The results of the thyroid uptake and scan in most GD patients showed homogeneity that was associated with high uptake, while the thyroiditis group showed heterogeneity that was associated with less nuclear uptake, which is consistent with a previous study [24, 25].

Among patients with a clinical hyperthyroid presentation in our study, the GD incidence rate was 40%, which is five-times higher than the thyroiditis incidence rate; the incidence rate of subclinical GD was 13.6%. A previous study reported that the incidence of painless thyroiditis among thyrotoxic subjects was 0.5% using the same evaluation method [26]. Unlike GD, thyrotoxicosis signs and symptoms are generally milder in TMNG [27].

Our study showed similar TSH and anti-TPO levels in GD and TMNG patients, but a higher level of free T3/T4 in GD patients. Thus, anti-TPO levels cannot be considered a differential diagnostic marker between GD and TMNG; rather, they can be considered a marker of autoimmunity [28].

Higher thyroglobulin antibody levels were found in patients with Graves’ ophthalmopathy than in patients without this ophthalmopathy [29]. However, current study showed that eye manifestations were nearly similar in the GD and TMNG groups.

The American College of Radiology Appropriateness Criteria Thyroid Disease stated that thyroid scanning as a diagnostic tool is not appropriate in the following cases: hypothyroid state, euthyroid state with palpable nodule but no goiter, and preoperative evaluation of thyroid cancer in euthyroid patients [30]. Despite these recent guidelines, our study showed that thyroid scans are inappropriately ordered for 8.2% of patients who were euthyroid and for 3.3% of patients who were hypothyroid at the time of the scan. Our study also showed that 4.3% of the thyroid scans showed normal results, 2% showed cold nodules, and 4.3% showed autonomous nodules. The physician’s inappropriate use of thyroid scans could be a consequence of routine workup for thyroid nodules having become a habit [31].

## Conclusion

GD is the most common form of hyperthyroidism, and it affects young females most frequently. Inappropriate thyroid uptake and scan use was reported in approximately 10% of patients, and 25% of patients used carbimazole during the uptake and scan. Thus, better patient education is needed to minimize scan result inaccuracies.

## Limitations

The study was conducted in a single tertiary care center (Al-Hada Hospital for Armed Forces in Taif, Saudi Arabia) and the sample size was limited to the number of patients who underwent ultrasonography and ^99m^Tc-pertechnetate thyroid scan during a 4-year period (2016–2019). Measurement of thyrotropin receptor antibodies (TRAb) was not available in our center during the study period.

## Supplementary information

**Additional file 1: Figure S1.** The use of carbimazole during the thyroid uptake and scan according to the uptake and scan diagnosis. **Figure S2.** Percentage of the ordered thyroid uptake and scan per year.

## Data Availability

The datasets used in this study are available from the corresponding author upon request.

## Abbreviations

GD: Graves’ disease
TMNG: Toxic multinodular goiter
TRAb: Thyroid receptor antibodies
Anti-TPO: Anti-thyroid peroxidase antibody
CRP: C-Reactive protein
ESR: Erythrocyte sedimentation rate
TSH: Thyroid stimulating hormone
FT4: Free thyroxine
FT3: Free triiodothyonine
HLA: Human leukocyte antigen
