# Supplementary material for: Scintigraphy evaluation of hyperthyroidism and its correlation with clinical and biochemical profiles
Source: BMC Res Notes. 2020 Jul 6;13:324. doi: 10.1186/s13104-020-05164-5 (PMC7339512; doi:10.1186/s13104-020-05164-5)
Supplement: Supplementary file 1 — Additional file 1: Figure S1. The use of carbimazole during the thyroid uptake and scan according to the uptake and scan diagnosis. Figure S2. Percentage of the ordered thyroid uptake and scan per year. [file 13104_2020_5164_MOESM1_ESM.pdf]

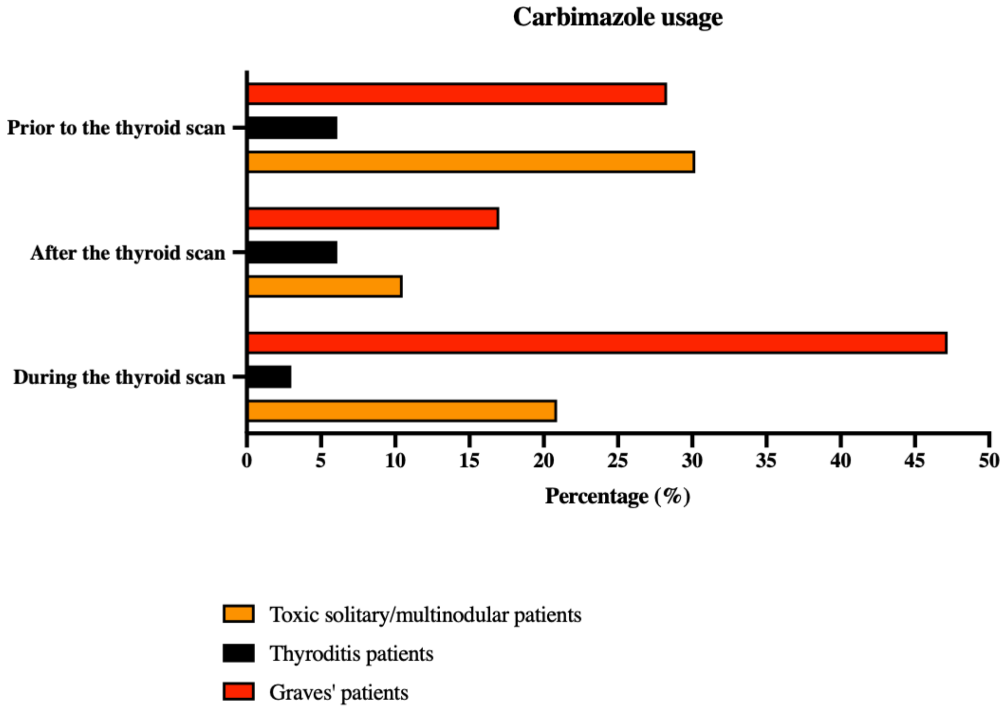

Figure.1 The use of carbimazole during the thyroid uptake and scan according to the uptake and scan diagnosis.

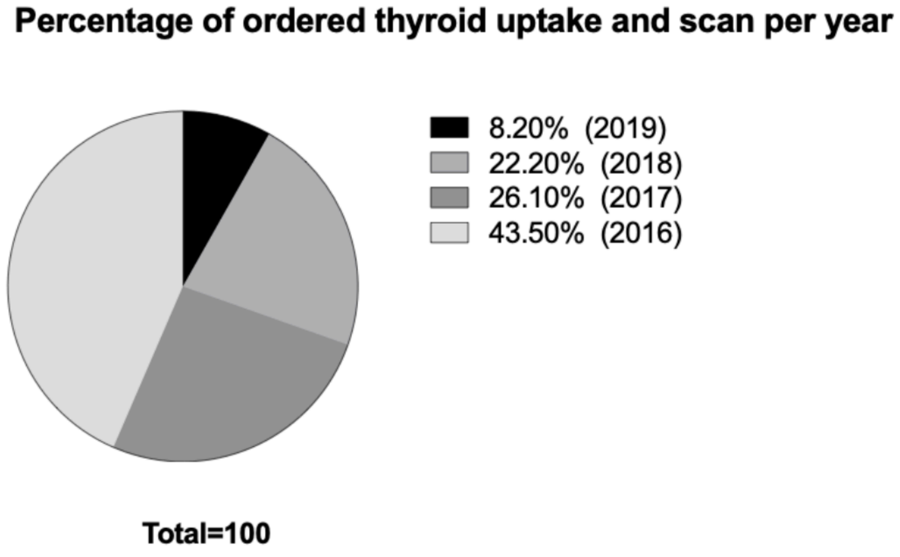

Figure.2 Percentage of the ordered thyroid uptake and scan per year.
